# Improvement of telescope resolution using a diffractive phase modulater

**DOI:** 10.1038/s41598-019-39804-z

**Published:** 2019-03-05

**Authors:** Yuxiang Wen, Kunpeng Wang, Dengfeng Kuang

**Affiliations:** 1 Institute of Modern Optics, Nankai University, Tianjin, 300350 China; 2 Beijing Institute of Tracking and Telecommunications Technology, Beijing, 100094 China

## Abstract

Metasurface, fluorescent microscopy and scanning near-field optical microscopy can improve the resolution of microscopes remarkably, while the resolution of the telescope remains unimproved constrained by its giant objective lenses and distant targets. Here we put forward a way to raise the resolution of telescopes simply by adding a binary optical thin surface around its focal plane. Simulation results show that the surface can raise the image quality in the Cassegrain and Kepler telescope. By nano-lathe, we fabricated a designed binary surface and experiment it in the Kepler telescope. The results are consistent with those of simulation results. More details of the calibrated target are resolvable on the image plane after applying the binary optical surface. It proves that the binary optic surface can make contribute to the resolution of the telescope, thus is beneficial in astronomy, military surveillance field.

## Introduction

Telescope is one of the most important optical instruments and it can be used in a variety of fields such as photography, scouting and space exploration. Since the invention of the telescope in the 17th century, people have made great efforts to improve the performance of the telescope. Achromatic lens^1^ and parabolic mirrors^2,3^ are introduced to telescopes to reduce the spherical and chromatic aberrations. However, the diffraction of light limits the minimum resolution in the forms of the Rayleigh criterion as 1.22 *λ*/D^4^, where *λ* denotes the incident wavelength and D refers to the diameter of the objective aperture. Increasing the diameter of the objective lens becomes an obvious way to increase the resolution, China has built a 500-meter aperture spherical telescope to explore the deep space^5^. Aperture-synthesis technique is an alternative way to enlarge the aperture diameter. The Very Large Array (VLA) in New Mexico of America comprises twenty-seven 25-meter radio telescope^6^. Adaptive optics^7,8^, Fourier telecopy^9,10^ and low-noise antenna^11^ are adopted to improve the signal-noise ratio (SNR) at different signal wavelengths. As the aperture becomes larger and the standard of the receiver becomes higher, the complicity and cost of a telescope skyrockets as well.

To cross the diffraction limit barrier and enhance the image quality, researchers presented super-oscillation technique^12,13^, fluorescent image microscope^14,15^, and evanescent wave detection microscopy using different kinds of probes^16^. The super-oscillation lens trades the light intensity with higher resolution. In 2009, Fumin Huang reached 0.61 diffraction limit by using a purposely-designed mask^17^. The evanescent wave mainly contributes to the image in two distinct ways, scans the evanescent wave like scanning near-field optical microscopy (SNOM) and using negative refractive index material to guide the evanescent wave such as super lens^18^. When the incident light is 600 nm, stimulated emission depletion microscopy (STED) has reached 20 nm resolution^19^ and structured illumination (SIM) is able to discover 120 nm particle in its field of views^20^ and stochastic optical reconstruction microscopy (STORM)^21^ or photoactivated localization microscopy (PALM) are able to distinguish particles with a 30 nm gap^22^. However, these super resolution methods mentioned above are difficult to apply to telescope because the fact that the light signal of a distant object is too faint to bear the loss of super oscillation lens and the aperture of the lens is excessively huge for a subwavelength structure in the metamaterial lens or super lens. In addition, the distant target is unlikely to dye with fluorochrome. Nevertheless, improving the resolution of the telescope is still a priority demand of distant target detection.

Diffractive optics has distinct dispersion character in contrast with traditional lens. Moreover, multiple freedom degrees of the sag provide diffractive optical element (DOE) with strong ability to correct the aberration of an optical system. Researchers have validated its on-axis and off-axis wavefront control ability in membrane mirrors^23,24^. Based on this consideration, diffractive optical element has been widely used to correct the aberration in optical image system. Ball Aerospace and Technologies Corp demonstrate a large aperture diffractive transmissive telescope^25^. DOE brought new opportunities for telescope with lighter weight, lower cost and easier fabricated surface sag. In addition, experiments have been made to prove the powerful aberration compensation ability of the DOE^26^. However, the telescope used in many scenarios are still composed of traditional lens or mirrors, improving the resolution of these telescopes without changing the basic configuration of them has significant meaning. for the sake of weight load and the image quality, here we propose a method to increase the resolution of telescopes without any adjustment to the objective lens or target preprocess, we add a diffractive optic element to the focal plane of the telescope to modulate the wavefront and correct the aberration. Both the simulation and the experiment show that the DOE we designed can make contributes to the resolution of the telescopes we used.

## Results

### Simulation of DOE in reflective telescope

**Figure 1:**
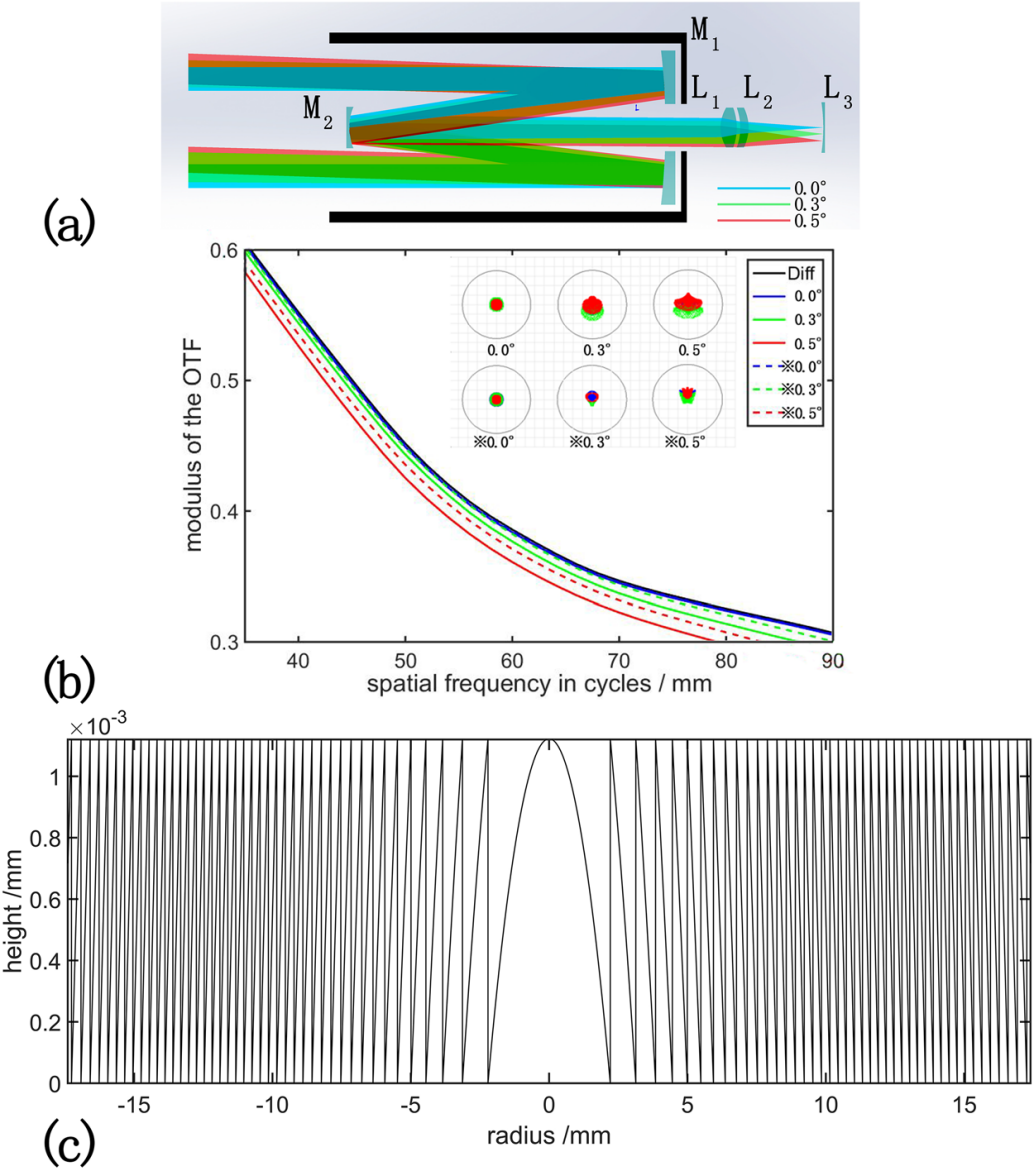
The performance of DOE in an optimized Cassegrain telescope (**a**) Schematic view of the telescope, there are two reflective mirrors (M1 and M2) as the objective lenses, two achromatic lenses (L1 and L2) and a Smyth lens (L3) as the eyepieces of the telescope. (**b**) The MTF and the spot diagram of the system. The content on the upper-right box is the legend of the MTF curves, the dash lines represents the MTF curves after applying the DOE. Red, green and blue represent three wavelengths of 486 nm, 587 nm and 656 nm in the upper-right spot diagram respectively and ⋇ means the effect after applying the DOE. (**c**) The cross section of the surface sag of the DOE in this system.

Most telescopes have multiple lenses in focal plane, such as the Smyth lens or Barlow lens. These lenses are served as field flatting and aberration correction^27,28^. Freeform lenses are designed to improve the resolution of telescopes^29^. However, these lenses are thick and heavy, and the sag of freeform lens is generally hard for fabrication due to the high order nonlinear term of the sag equation. Flat DOE is thin, light, and famous for its great ability to shaping and homogenizing the light beam. All the characters mentioned above make DOE a suitable element for wavefront correction. Firstly, as reflective telescope is the most common astronomical telescope, we use a Cassegrain telescope model as the initial structure of the system [Fig. 1(a)]. We also add an achromatic lens (L1 and L2) and a Smyth lens (L3) as the eyepiece of the system to rectify the aberration of the system. After we optimize the system, the first mirror has a radius of −1200 mm and the conic rate is −1.038, the second mirror has a radius of −394.737 mm and the conic rate is −3.004. The eye piece simulated in our system consists of two lenses, the first lens is make of 5 mm N-PSK53 with a radius of 225.038 mm and −1940.972 mm. the second lens is make of 5 mm SF1 with a radius of 11970 mm and 457.65 mm. The spot radius of the central field of view (FOV) reaches 1.4 *μ*m and the Seidel coefficients of the system (S1, S2, S3, S4) are all below 10^−3^, the spot radius and the modulate transfer function (MTF) of the system are shown in Fig. 1(b).

Figure 1(a) shows the simulated optical path of the reflective telescope, the incident light angle magnified by objective lens (M1 and M2) passes through two lenses (L1 and L2) to modify the spherical, coma and astigmatic aberration. Near the image plane, a 3-mm-thick lens is used to flat the field and correct the distortion and curvature aberration caused by the former system. Seidel aberration coefficients of at the image plane are 0.000396(S1), 0.000104(S2), −0.000014(S3), 0.000235 (S4) and −0.014364(S5). The MTF curves are presented in Fig. 1(b) as the straight line and the spot diagram is shown in the first row of spot diagram at the top of Fig. 1(b). The resolution of the system is good enough for practical detection. To test the performance of the DOE, we replace L3 with a 0.1 mm-thick DOE. The surface sage of the binary element we use are:

1 $$Z(r)=\frac{M\lambda }{2\pi (n-\mathrm{1)}}(\sum _{i=1}^{N}{A}_{i}\frac{{r}^{2i}}{\rho }\,mod\,2\pi )$$

where Z is the height of the sag, r is the radius of the sag, *λ* is the wavelength of the incident light, n is the refractive index, M is the diffraction order, N is the number of the terms, *ρ* is the normalized radial aperture coordinate and *A*_*i*_ is the i coefficient on the *i*^*th*^ polynomial term, mod means modulo operation. In this simulation, M equals to 1. *λ* equals to 550 nm, N equals to 2, n equals to 1.49 and *A*_1_ equals to −390.993, *A*_2_ equals to 118.548 with the normalized radius *ρ* equals to 17.397 mm. The sag of the DOE is shown in Fig. 1(c). As we can see in this picture, the MTF curves of the original system approximate the diffraction limit when the field angle approaches 0°. While after we apply the DOE in this system, the MTF curves rises in all FOVs, and the increase is extremely obvious in off-axis field angles. The red dashed line in Fig. 1(b) is apparently higher than the straight red line. From the spot diagram on the upper part of Fig. 1(b), we can also see that the original system is optimized to a good level, and the DOE shrink the spot diagram and reduce the chromatic aberration in a further step, the spot radius of ⋇ 0.5° is smaller than that of 0.5°. Seidel coefficients of the system with DOE are 0.000331(S1), 0.000015(S2), −0.000082(S3), 0.000052 (S4) and −0.014528(S5), the spherical aberration, coma and field curvature are sharply decreased.

### DOE in Kepler telescope

#### Simulation

**Figure 2:**
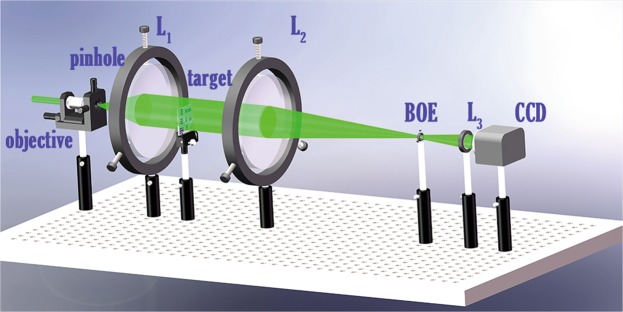
The schematic view of the refractive telescope system, from left to right are the micro-objective, pinhole, collimator lens (L1), calibration target objective of the telescope (L2), DOE, eyepiece of the telescope (L3) and a CCD.

We also check the performance of the DOE in a simple Kepler telescope as a proof-of-principle experiment. The DOE is given as aberration compensation element in the focal plane of the telescope. The system layout shows in Fig. 2.

**Figure 3:**
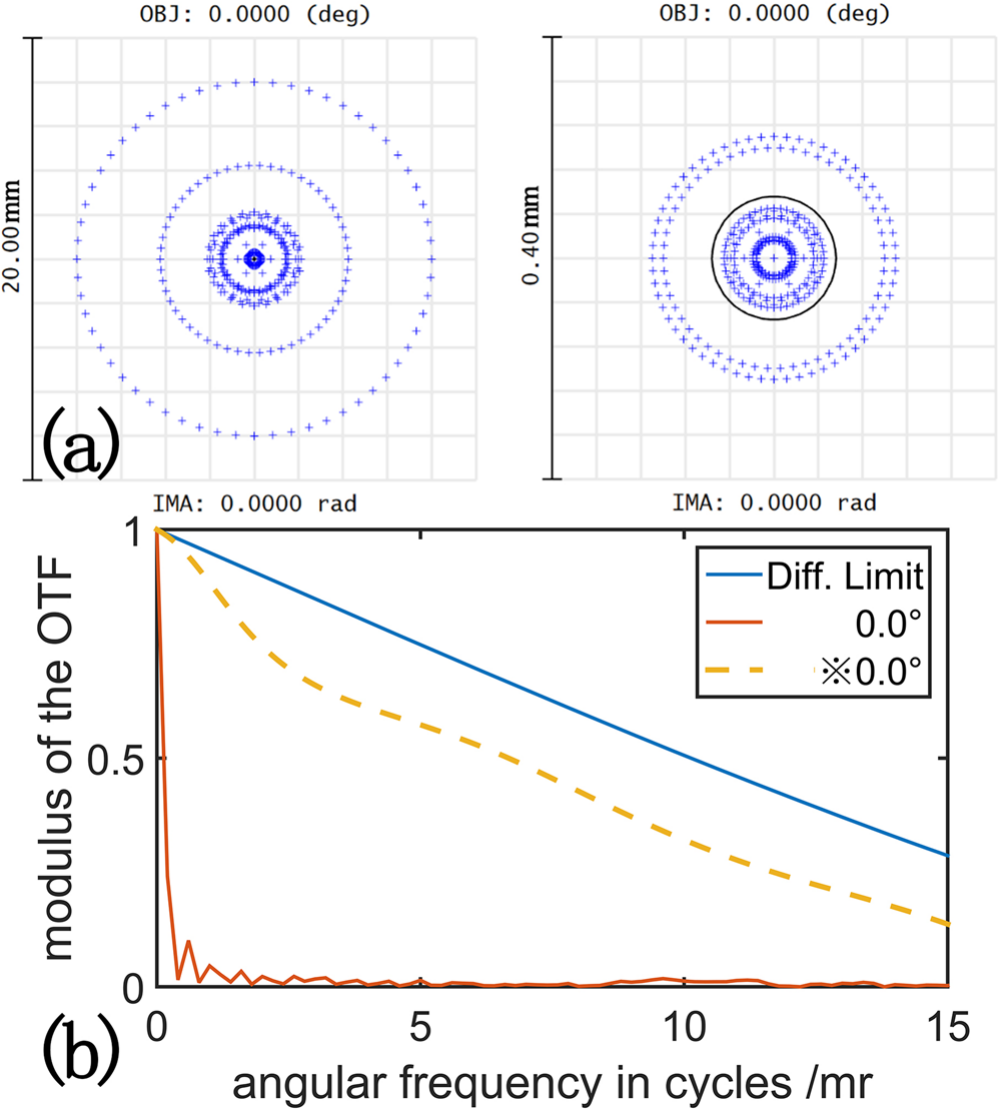
(**a**) The spot diagram of the system before (left) and after (right) applying the DOE. (**b**) The MTF of the system, the blue line is the MTF of the diffraction of the system; yellow dash line and red line are the MTF with and without the DOE.

The incident light (532 nm) passes through the micro-objective and the pinhole to become a point light source, and then a lens is served as a collimator to generate parallel light. The parallel beam lighting in the telescope across a calibration target. We use two plano-convex lenses as the objective and the eyepiece of the telescope, and a DOE is set in the focal plane of the telescope to calibrate the wave front. Finally, a CCD capture the emerging light. The radius of the objective lens is 156 mm with a diameter of 76.2 mm and the radius of the eyepiece lens is 31 mm with a diameter of 30 mm, the material of the lenses are both BK7 and the angular magnification rate of this system is 5. Due to the dispersion and the stray light of the system, the geometric spot radius of the simulated system is 8 mm when the diameter of the entrance pupil is 68.58 mm, moreover the MTF curves is extremely low as shown in Fig. 3(b). When we add a DOE lens in the focal plane, the geometric spot radius is shrink to 0.174 mm with the same entrance pupil, and the MTF curves is much more closer to the diffraction limitation [Fig. 3]. The S1 of the Seidel coefficients drops from 0.184 to 0.163. The sag equation is the same as we used in the Cassegrain telescope, while *λ* equals to 532 nm, *ρ* equals to 3.574 mm.

#### Fabrication and experiments

**Figure 4:**
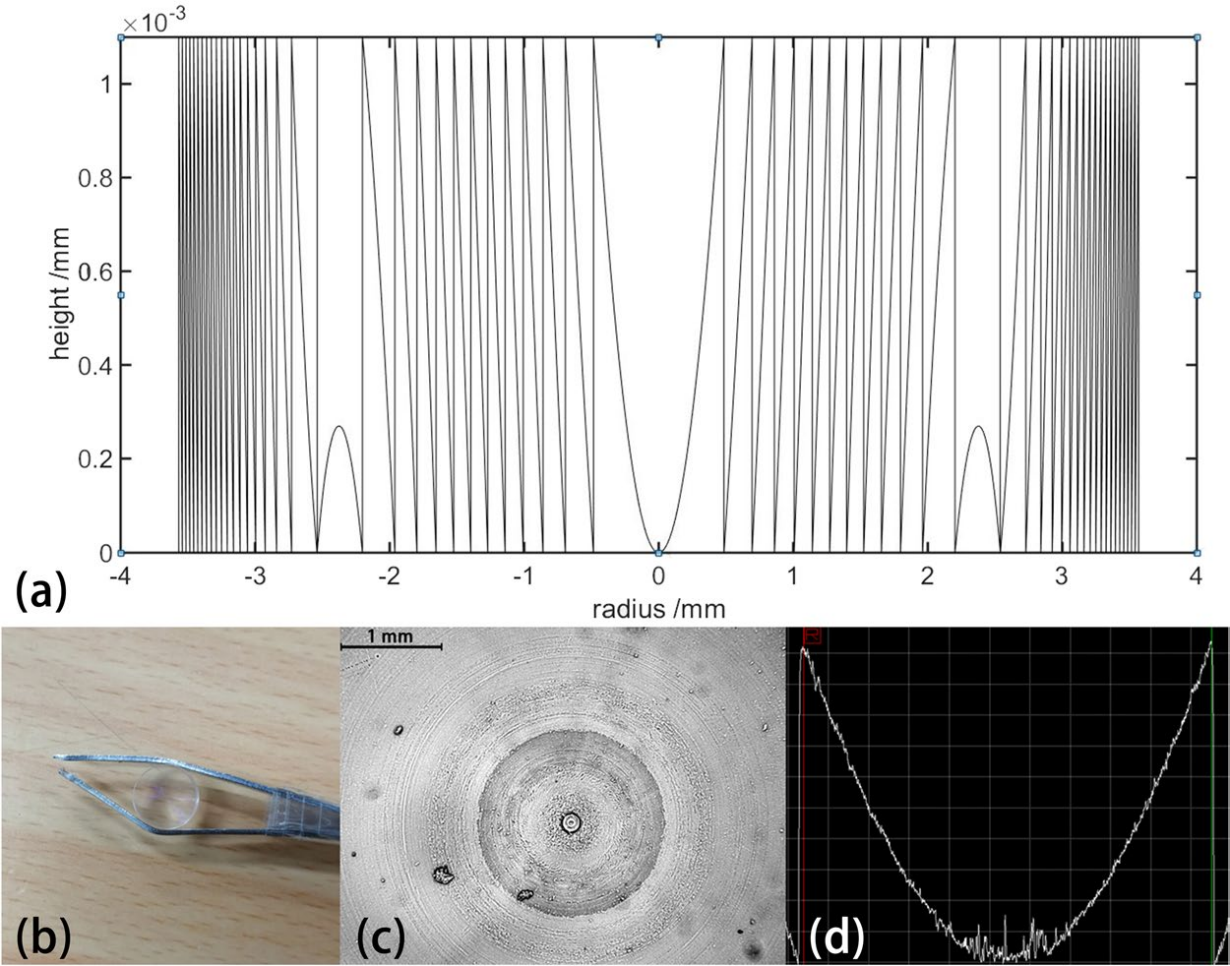
(**a**) The cross section of the surface sag of the DOE. (**b**) is the practice picture of the DOE composed of PMMA. (**c**) is the image of the DOE in an inverted microscope. (**d**) is the measurement result of the center sag of the DOE using a surface profiler (the width of the grid is 100 *μ*m and the height of the grid is 100 nm).

For fabrication convenience, we choose N equals to 2 and *A*_1_ equals to −349.179, *A*_2_ equals to 396.199, indicating that the highest order of the sag is four. The sag of the DOE is shown in Fig. 4(a). The sag surface comprises two parts, in the middle of the DOE is a convex sag and the side of the DOE has a concave shape. The change of the sag is adapted to the on axis and off axis aberration in this system. Considering the difficulty of fabrication, we choose PMMA as the material of DOE. The incident wavelength is 632.8 nm and the refractive index of the PMMA in the wavelength is 1.49. The thickness of the surface is 1.09 *μ*m. We use nanolathe to fabricate the surface, and add a 5-mm-thick PMMA as the substrate to clamp the element when fabricating the surface. Fabricated DOE and its measurement results are shown in Fig. 4. The samples (diameter 7.1 mm) were fabricated on a PMMA substrate (refractive index n = 1.49) at a max tuning depth 1.09 *μ*m through nano lathe. kinetic accuracy of the lathe rail is 10 nm. From Fig. 4(c,d), we can note that the size of the DOE is small and the weight of it is 0.26 g. From the measurement result we can see that the sag of PMMA processed by nanolathe shows good agreement with the simulation. There is no notable step at different radius, so the diffraction efficiency remains at a high level.

**Figure 5:**
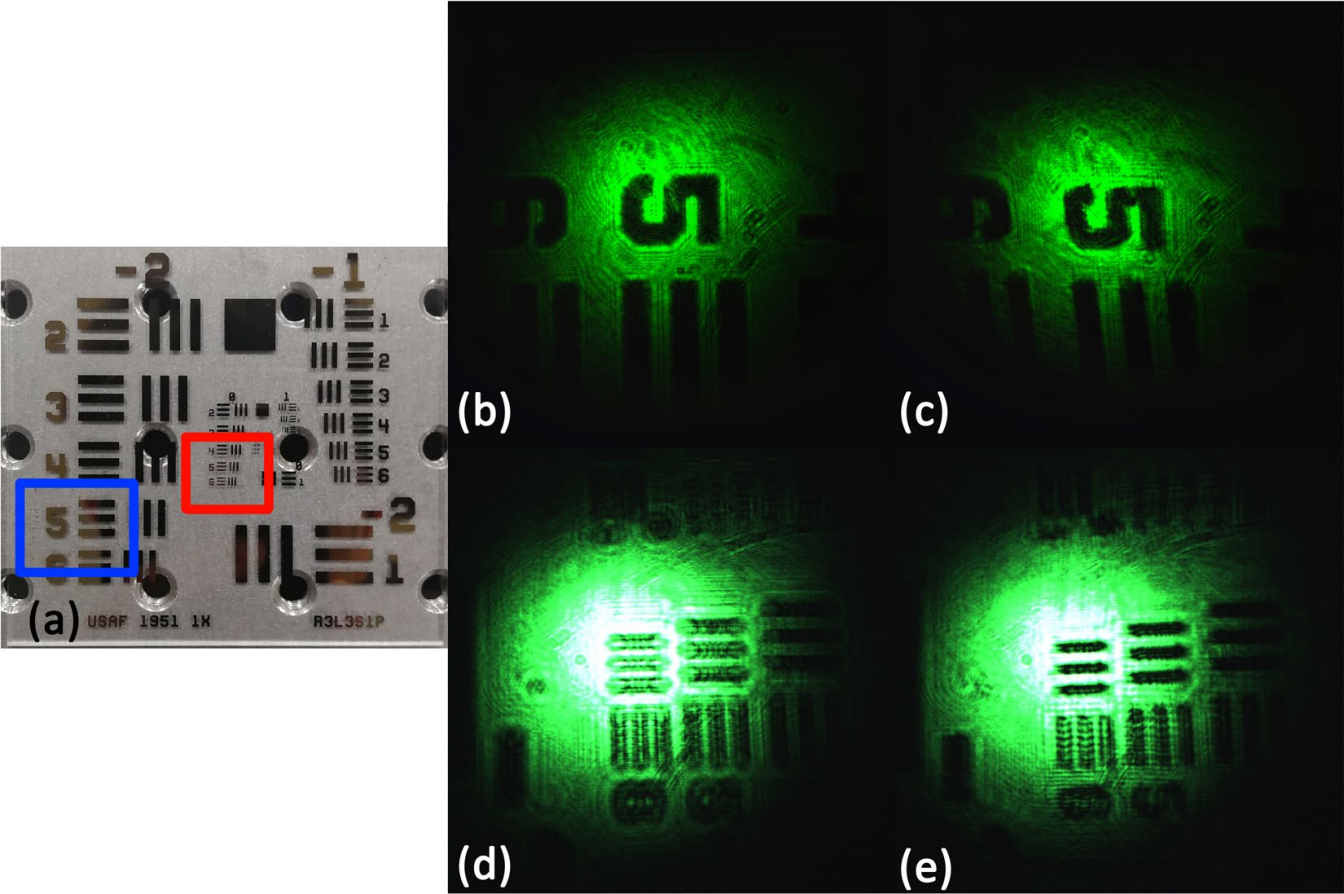
The experimental result of the image of the telescope system, (**a**) is the calibration target and the background circle is the location hole of the optical table, whose diameter is 8 mm, the blue square represents the area of (**b**,**c**), the red square represents the area of (**d**,**e**). (**b**,**d**) are the images without the DOE and (**c**,**e**) are the images after using the DOE.

We use this PMMA element in the focal plane of the telescope and the results are shown in Fig. 4. In this experiment, the calibration target used in this system is around 4 mm and the gap between the black stripes is around 0.3 mm, which are shown in Fig. 5(a). From the image captured by CCD [Fig. 5(b)], we can see that the image of the calibration target is not distinguishable after magnified by the telescope system. Moreover, the gaps between the stripes are completely unresolved at the imaging plane of the system. However, after applying the DOE in the focal plane [Fig. 5(c)], the horizontal stripes in the middle field are clear and the other stripes are distinguishable as well. The gap between each stripe is resolvable, which means that the resolution of the system is remarkably improved. However, distortion appeared as the image becomes distinct after insert the DOE.

## Discussion

In summary, we simulate the performance of DOE as a field-flatting lens in the eyepieces of a well-designed reflective telescope. The spot radius of the system at different FOVs is greatly shrined and the MTF of the system is increased in all three FOVs and has an obvious increment at off-axis FOV, which indicates that the DOE can increase the performance of a reflective telescope. We also conduct a prove-of-principle experiment using a simple Kepler telescope. After the simulation in Zemax, we fabricate the DOE utilizing nanolathe, the fabricated DOE is made up of PMMA and has a 5 mm substrate of the same material. We contrast the image of a calibration target before and after inserting the DOE in the focal plane of the Kepler telescope. The gap between the stripes in the calibration target is resolvable after inserting the DOE, and the shape of the stripes becomes clearer. We conclude that a well-designed DOE can improve the resolution of the telescope. Considering the volume and weight of the DOE are much smaller than optical lenses, the DOE we made can make a significant contribution to military exploration and space exploration. However, when we applying the DOE in the system, some distortion appeared in the image, we consider this problem will be alleviated if we add the order of the sag.

## Methods

### DOE fabrication

The DOE (diameter 7.1 mm) were fabricated on a 4-mm-thick PMMA substrate (n = 1.49)at a maximum tunning depth 1.09 *μ*m through nanolathe (Nanotech 250 UPL).The kinetic accuracy of the lathe rail is lower than 100 nm per 75 mm in diameter, and the surface altitude accuracy is lower than 2 nm.

### Experimental setup

Fig. 2 shows the schematic of the optical experimental setup, including a laser(532 nm), a pin-hole(diameter 0.1 *μ*m), an objective lens(magnification rate = 25, numerical aperture = 0.4, working distance = 0.17), a test target(THORLABS R3L3S1P), a collimator lens and an objective lens(L_2_, diameter = 76.2 mm, f = 300 mm) and an eyepiece(L_3_, diameter = 30 mm, f = 60 mm) and a CCD camera (Micron MT9P031, 2592*1944, pixel size 2.22 *μ*m). The incident light passes through the pin-hole, an objective lens and then collimated as an parallel ray. The collimated light incidents into the objective lens L_2_, L_3_ is settled 360 mm away from L_2_ and the DOE we fabricated is settled 330 mm away from L_2_.

## References

[1] Lessing NVW (1969). Five-or six-lens achromatic anamorphotic telescopes. Applied optics.

[2] Schmidtke G, Henneberg P, Hager KH, Busch F, Reinhardt D (1980). Parabolic telescope and spectrometer combination. Applied Optics.

[3] Díaz-Uribe R (2000). Medium-precision null-screen testing of off-axis parabolic mirrors for segmented primary telescope optics: the large millimeter telescope. Applied Optics.

[4] Barakat R (1965). Rayleigh wavefront criterion. Josa.

[5] Nan R (2006). Five hundred meter aperture spherical radio telescope (fast). Science in China.

[6] Napier PJ, Thompson AR, Ekers RD (1983). The very large array: Design and performance of a modern synthesis radio telescope. Proceedings of the IEEE.

[7] Hardy, J. W. Book review: Adaptive optics for astronomical telescopes/oxford u press, 1998. *Physics Today* **53** (2000).

[8] Wang JY, Markey JK (1978). Modal compensation of atmospheric turbulence phase distortion*. *Optical Society of America, Journal, vol. 68, Jan. 1978, p. 78–87*. Research supported by the General Dynamics Corp..

[9] Tegmark, M. & Zaldarriaga, M. The fast fourier transform telescope. *Physical Review D Particles and Fields* **79** (2009).

[10] Lei D, Xinyue L, Xudong L, Peifeng W, Shuhai Y (2012). Improvement of performance and analysis of results of field experiments of fourier telescope. Acta Optica Sinica.

[11] Gawande R, Bradley R (2011). Towards an ultra wideband low noise active sinuous feed for next generation radio telescopes. IEEE Transactions on Antennas and Propagation.

[12] Rogers ET (2012). A super-oscillatory lens optical microscope for subwavelength imaging. Nature Materials.

[13] Wang C (2015). Super-resolution optical telescopes with local light diffraction shrinkage. Sci Rep.

[14] Fernándezsuárez M, Ting AY (2008). Fluorescent probes for super-resolution imaging in living cells. Nature Reviews Molecular Cell Biology.

[15] Shtengel G (2009). Interferometric fluorescent super-resolution microscopy resolves 3d cellular ultrastructure. Proceedings of the National Academy of Sciences of the United States of America.

[16] Kumar A, Tabib-Azar M (1999). Comments on evanescent microwaves: a novel super-resolution noncontact nondestructive imaging technique for biological applications [with reply]. IEEE Transactions on Instrumentation and Measurement.

[17] Rogers, E. T. F., Zheludev, N. I. & Chad, J. E. Super-oscillatory lens apparatus and methods (2015).

[18] Weisenburger S, Sandoghdar V (2015). Light microscopy: an ongoing contemporary revolution. Contemporary Physics.

[19] Göttfert F (2013). Coaligned dual-channel sted nanoscopy and molecular diffusion analysis at 20 nm resolution. Biophysical Journal.

[20] Gustafsson MG (2000). Surpassing the lateral resolution limit by a factor of two using structured illumination microscopy. Journal of Microscopy.

[21] Jia S, Vaughan JC, Zhuang X (2014). Isotropic 3d super-resolution imaging with a self-bending point spread function. Nature Photonics.

[22] Hess (2006). Ultra-high resolution imaging by fluorescence photoactivation localization microscopy. Biophysical Journal.

[23] Gruneisen MT, Martinez T, Lubin DL (2002). Dynamic holography for high-dynamic-range two-dimensional laser wavefront control. Proc Spie.

[24] Gruneisen MT, Dymale RC, Rotge JR, Lubin DL (2002). Near-diffraction limited compensated imaging and laser wavefront control with programmable diffractive optics. Proceedings of SPIE - The International Society for Optical Engineering.

[25] Atcheson, P. *et al*. Moire: ground demonstration of a large aperture diffractive transmissive telescope. In *Spie Astronomical Telescopes* + *Instrumentation*, 91431W (2014).

[26] Gruneisen, M. T., Desandre, L. F., Dymale, R. C., Rotgé, J. R. & Lubin, D. L. Compensated telescope system with programmable diffractive optic. *Optical Engineering* **44** (2005).

[27] Panchuk VE, Chuntonov GA, Naidenov ID (2014). Main stellar spectrograph of the 6-meter telescope. analysis, reconstruction, and operation. Astrophysical Bulletin.

[28] Scharmer GB, Brown DS, Pettersson L, Rehn J (1985). Concepts for the swedish 50-cm vacuum solar telescope. Appl Opt.

[29] Gautam S, Gupta A, Singh GS (2015). Optical design of off-axis cassegrain telescope using freeform surface at the secondary mirror. Optical Engineering.

